# The complex interplay of hypoxia and sleep disturbance in gray matter structure alterations in obstructive sleep apnea patients

**DOI:** 10.3389/fnagi.2023.1090547

**Published:** 2023-03-31

**Authors:** Jing Wang, Yezhou Li, Lirong Ji, Tong Su, Chaohong Cheng, Fei Han, Daniel J. Cox, Erlei Wang, Rui Chen

**Affiliations:** ^1^Department of Respiratory, The Second Affiliated Hospital of Soochow University, Suzhou, China; ^2^Department of Sleeping Center, The Second Affiliated Hospital of Soochow University, Suzhou, China; ^3^School of Biological Sciences, University of Manchester, Manchester, United Kingdom; ^4^Department of Radiology, The Second Affiliated Hospital of Soochow University, Suzhou, China; ^5^Division of Psychology, Communication, and Human Neuroscience, School of Health Sciences, Faculty of Biology, Medicine and Health, University of Manchester, Manchester, United Kingdom

**Keywords:** obstructive sleep apnea, hypoxia, sleep disturbance, gray matter, structural equation models

## Abstract

**Background:**

Obstructive Sleep Apnea (OSA) characteristically leads to nocturnal hypoxia and sleep disturbance. Despite clear evidence of OSA-induced cognitive impairments, the literature offers no consensus on the relationship between these pathophysiological processes and brain structure alterations in patients.

**Objective:**

This study leverages the robust technique of structural equation modeling to investigate how hypoxia and sleep disturbance exert differential effects on gray matter structures.

**Methods:**

Seventy-four Male participants were recruited to undergo overnight polysomnography and T1-weighted Magnetic Resonance Imaging. Four structural outcome parameters were extracted, namely, gray matter volume, cortical thickness, sulcal depth, and fractal dimension. Structural equation models were constructed with two latent variables (hypoxia, and sleep disturbance) and three covariates (age, body mass index, and education) to examine the association between gray matter structural changes in OSA and the two latent variables, hypoxia and sleep disturbance.

**Results:**

The structural equation models revealed hypoxia-associated changes in diverse regions, most significantly in increased gray matter volume, cortical thickness and sulcal depth. In contrast, sleep disturbance. Was shown to be largely associated with reduce gray matter volume and sulcal depth.

**Conclusion:**

This study provides new evidence showing significant effects of OSA-induced hypoxia and sleep disturbance on gray matter volume and morphology in male patients with obstructive sleep apnea. It also demonstrates the utility of robust structural equation models in examining obstructive sleep apnea pathophysiology.

## 1. Introduction

Obstructive sleep apnea (OSA) is a highly prevalent multisystem chronic disease, which predisposes patients to diseases including hypertension, diabetes mellitus, stroke, emotional disorders and cognitive impairments such as memory and attention deficits (Chen et al., 2011; Rosenzweig et al., 2015; Benjafield et al., 2019; Bubu et al., 2020; Gottlieb and Punjabi, 2020). The main characteristic of OSA is the repetitive airway collapse, partially reducing or completely blocking the pharyngeal airflow despite respiratory efforts leading to chronic intermittent hypoxia (Dewan et al., 2015). Frequent blocking also may lead to sleep disturbance, manifested by a decrease in the deeper stages of sleep.

Neuroimaging studies have shown that OSA may be associated with gray matter structural alterations. A meta-analysis revealed a clear decrease in gray matter volume (GMV) in middle aged untreated patients with OSA (Shi et al., 2017). However, recently, using more advanced neuroimaging measures, Baril et al. (2017) found a consistent positive relationship between markers of OSA severity (hypoxemia, respiratory disturbances, and sleep structure alteration) and gray matter hypertrophy and thickening, while another large sample study observed lower mean oxygen saturation during sleep was associated with atrophy of both cortical and subcortical brain areas (Marchi et al., 2020). Furthermore, Cross et al. (2018) and his colleagues reported more complex results, that is, hypoxia metrices were shown to be associated with decreased cortical thickness, while sleep disturbance with increased thickness. Although researchers have posited the presence of two counteracting mechanisms (One may indicate cellular damage, while the other may reflect transitory responses) to account for these seemingly contradictory results (Baril et al., 2021), no consensus on the exact pattern of OSA-induced gray matter changes can be drawn from the current literature (Baril et al., 2017; Cross et al., 2018; André et al., 2020; Marchi et al., 2020).

To characterize OSA severity, previous studies often used individual polysomnographic parameters (e.g., apnea-hypopnea index (AHI), mean oxygen saturation) to examine OSA-related gray matter changes. However, such single-parameter analyses are prone to producing spurious results due to the measurement error. Structural equation modeling (SEM) could be used to model the complex relationship between multiple inter-dependent variables in a multi-level variable structure. It permits the exploration of complex relationships among a number of potentially inter-dependent variables (Bentler and Stein, 1992). In the context of this study, using the SEM model accounts for and even takes advantage of the collinearity between variables by extracting the covariance between related variables as physiologically meaningful latent variables. Moreover, it allows for a more principled analysis of the OSA pathophysiological processes, as well as their potentially differential effects on the OSA brain.

Despite the popularity of GMV, it is a coarse measure that captures both a region’s surface area and cortical thickness, which might convey distinct pathophysiological implications (Fornito et al., 2008; Winkler et al., 2010). Therefore, the current study also included the use of Surface-Based Morphometry (SBM) to provide measures of cortical thickness and surface complexity. To quantify cortical complexity, fractal dimension (FD) utilizes the concepts of fractals to characterize the nested and hierarchical structures of the brain (Madan and Kensinger, 2016) Sulcal depth (SD), defined as the Euclidean distance between the central surface and an imaginary convex hull encompassing the surface mesh, has been shown to change significantly in various patient groups such as Parkinson’s disease (Wang et al., 2021). To our knowledge, there have not been studies investigating the cortical complexity in the OSA population.

In this study, we constructed an SEM model consisting of two latent variables—hypoxia and sleep disturbance, to examine the effects of these two physiological factors on gray matter structure. Based on previous studies, we hypothesize that there may be a mixture of effects for both hypoxia and sleep disturbance in affecting different regions of the OSA brain.

## 2. Participants and methods

### 2.1. Participants

This prospective study recruited 74 patients who presented with a primary complaint of snoring and were formally diagnosed with OSA at the sleep center of the Second Affiliated Hospital of Soochow University from August 2020 to September 2021. The OSA diagnosis was confirmed by polysomnography (PSG) with an AHI ≥ 5 (Kapur et al., 2017). Participants were between 25 to 60 years old (median age = 39 years, SD = 9.6 years) In terms of participant genders, as previous large-sample studies found that the clinical phenotype of female patients with OSA differs from that of male patients (Basoglu and Tasbakan, 2018; Bonsignore et al., 2019). Moreover, another study found more severe OSA-related white matter tract damages in female patients than their male counterparts with similar OSA severity (Macey et al., 2012). Therefore, given that OSA is a significantly male-dominant disorder (Young et al., 1993; Franklin and Lindberg, 2015; Theorell-Haglöw et al., 2018) and that the anticipated extent of gray matter structural change is small, the present study chose to include only male patients to reduce sample heterogeneity. Participants with a history of neurological, respiratory, or other medical conditions that might affect sleep were excluded. The participants gave informed consent, and the study protocol was approved by the Research Ethics Committee of the Second Affiliated Hospital of Soochow University, Suzhou, China (JD-LK-2018-004-02).

### 2.2. PSG

The participants underwent overnight, supervised, laboratory-based video polysomnography (PSG) using the Compumedics Grael multifunctional PSG monitoring system. Sleep staging and sleep-related respiratory analyses were scored manually by registered technician according to the AASM scoring criteria (Kapur et al., 2017). Apnea was defined as any airflow reduction greater than 90% that lasted longer than 10 s. Hypopnea was defined as >3% desaturation from pre-event baseline or arousal. The AHI was defined as the sum of the number of apnea and hypopnea per hour of sleep. Other measures included total sleep time (TST), sleep efficiency (SE), oxygen desaturation index (ODI), proportion of sleep time with SaO₂ < 90% (T90), minimum pulse oxygen saturation (MinSaO2), arousal index, and proportions of each sleep stage.

### 2.3. MRI

Magnetic Resonance structural images were collected using a T1-weighted magnetization-prepared rapid-acquisition gradient echo (MPRAGE) sequence on a 3 T Siemens Prisma MRI scanner. The voxel size was 1.0 × 1.0 × 1.0 mm^3^. The acquired structural MR images were bias-corrected, segmented, normalized, and 8 mm-smoothed using the Statistical Parametric Mapping (SPM12) toolbox^1^ and the Computational Anatomy Toolbox (CAT12, neuro-jena.github.io/cat/; Theorell-Haglöw et al., 2018) in MatLab (MathWorks, 2020). The regional average gray matter volumes of a total of 142 ROIs were extracted for each subject using the neuromorphometrics atlas.

**Figure 1:**
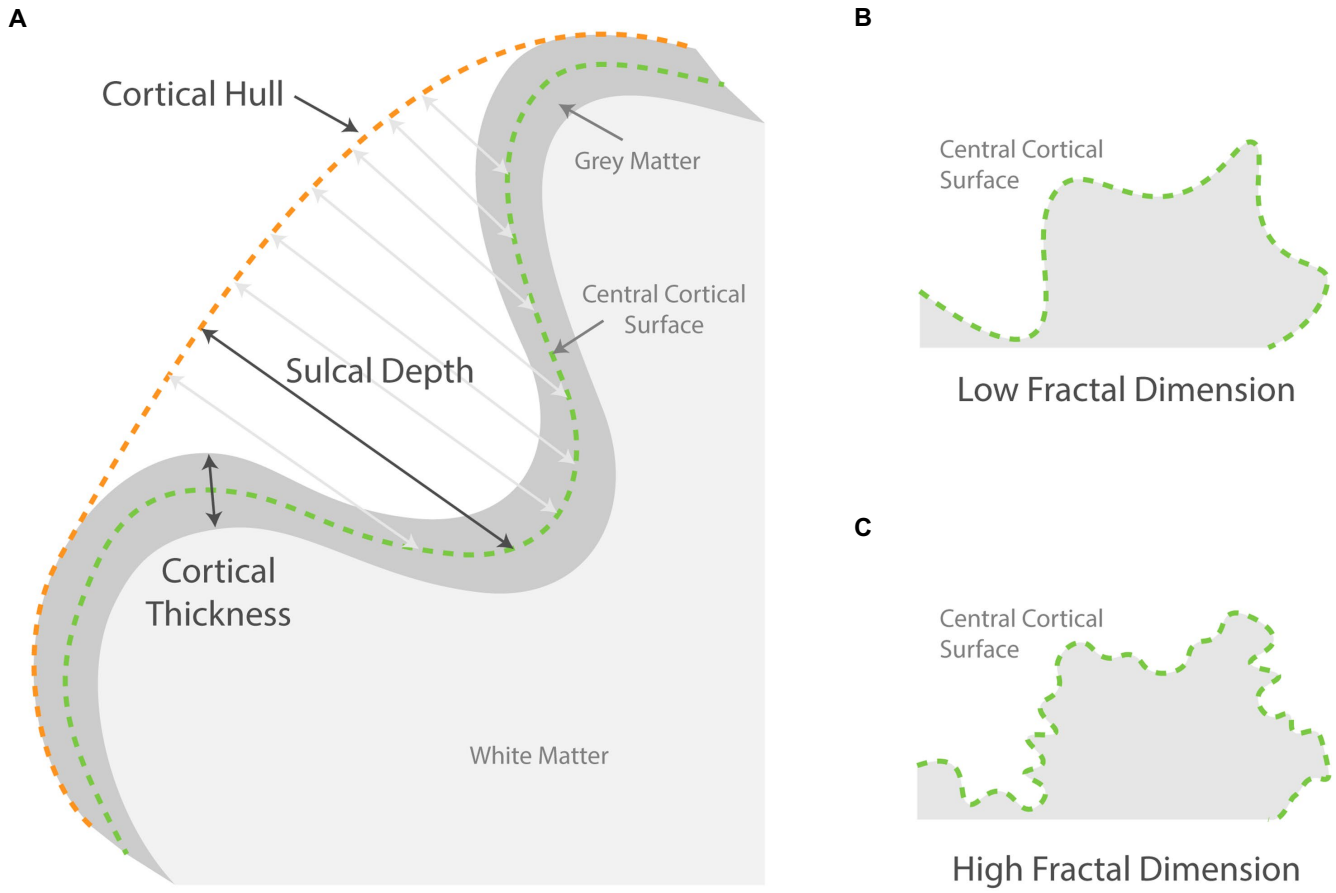
Schematic showing how surface-based parameters are measured. **(A)** Illustrates how sulcal depth at various points is measured from the central surface to the cortical hull. The cortical thickness is measured from gray matter outer surface to the inner surface; **(B,C)** compares two schematic surfaces with high and low fractal dimensions.

A separate CAT12 surface-based pipeline was used to produce a normalized surface mesh for each participant. Using the surface mesh, cortical thickness (Dahnke et al., 2013), sulcal depth, and fractal dimension were estimated (see Figure 1 for an illustration). The images of cortical thickness were smoothed using an isotropic Gaussian kernel of 15 mm and images of the complexity measures were smoothed using a kernel of 20 mm for the filter size to encompass both the sulcal fundus and gyral crown. Finally, mean values for cortical thickness, sulcal depth, and fractal dimension across the 72 surface ROIs were extracted using the Desikan-Killiany DK40 atlas (Desikan et al., 2006).

### 2.4. SEM

**Figure 2:**
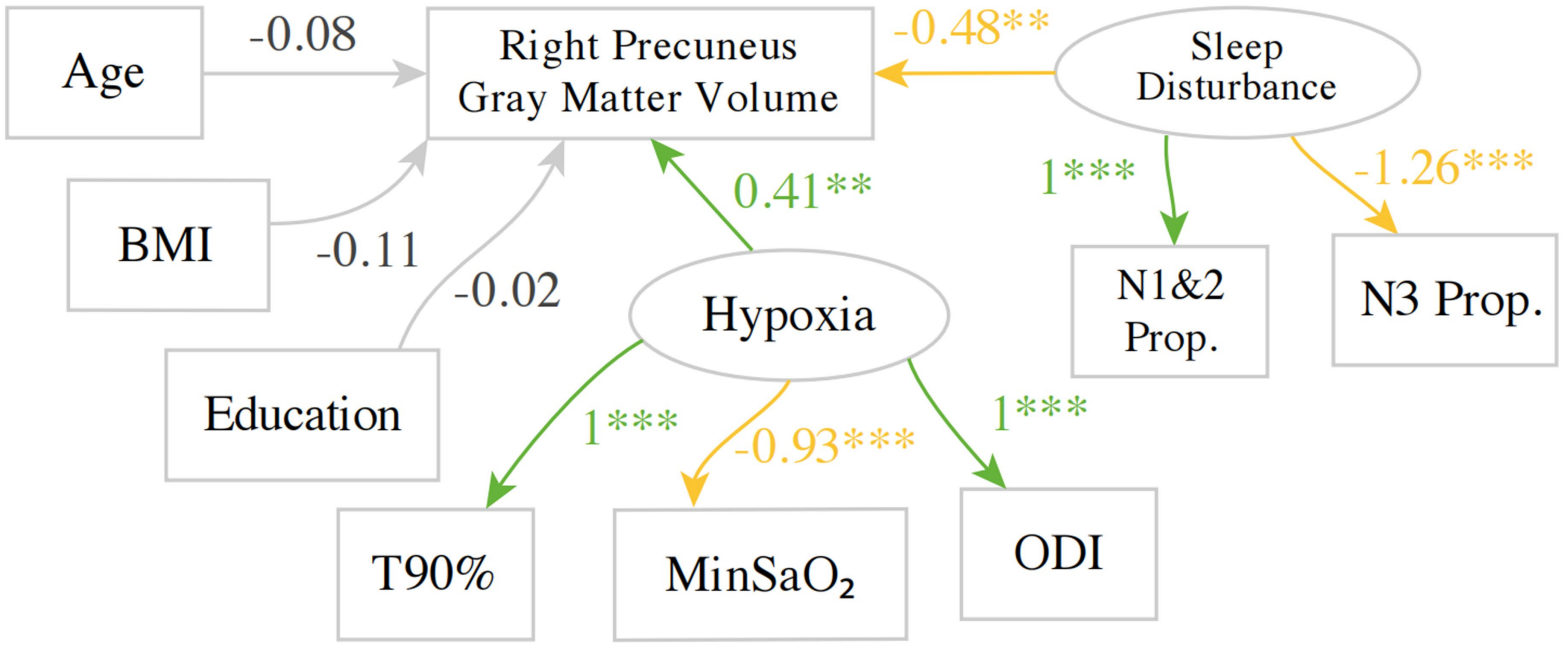
An example SEM model investigating the predictors for gray matter volume in the right precuneus. Green indicates a positive estimate, whereas yellow indicates a negative estimate. BMI, body mass index; T90%, Proportion Time with SaO₂ < 90%, MinSaO₂, Minimum pulse oxygen saturation; ODI, oxygen desaturation index; N1&2P, NREM 1 + 2 Sleep Proportion; N3P, NREM3 Sleep Proportion. **p < 0.01, ***p < 0.001.

Two latent variables (hypoxia and sleep disturbance) were included in the SEM model (see Figure 2). Hypoxia combined three indicator (observed) variables: minSaO₂, T90%, and ODI. The latter, sleep disturbance, was constructed from two negatively correlated indicator variables: proportion of sleep time in the NREM1&2 sleep and NREM3 sleep. It was decided that AHI should not be included as indicator variables as it is a measure of the apnea frequency, which is thus not directly measuring hypoxia or sleep disturbance. AHI also correlates strongly with indicator variables for both hypoxia and sleep disturbance, and thus its inclusion in the SEM model would lead to statistical difficulties in the model computations. The four ROI-based V/SBM measures were then entered individually into the SEM model as the outcome measure. Including the three covariates, age, body mass index (BMI), and education, the main regression equation for the SEM model was formulated as follows:


$$\begin{array}{l} OutcomeMeasure\sim Hypoxia+SleepDisturbance\\ \;\;\;\;\;\;\;\;\;\;\;\;\;\;\;\;\;\;\;\;\;\;\;\;\;\;\;\;\;\;\;\;\;\;\;\;\;\;\;\;\;\;\;\;\;\;\;\;\;+Age+BMI+Education \end{array}$$

where the Outcome Measure can be any of the 142 + 72 * 3 = 358 ROI-based parameter means. To account for the potential false discovery effect, *p* values were corrected using the Benjamini-Hochberg procedure. All statistical analyses apart from the MR image processing were carried out using the R statistical package (version 4.1.1).^2^

## 3. Results

### 3.1. Demographic, clinical, and sleep characteristics

Table 1 shows the clinical characteristics and PSG parameters of this study cohort. Patients with OSA exhibit hypoxia and sleep structural abnormalities as shown by PSG parameters. Data are represented by means and inter-quartile range (IQR).

**Table 1:** Descriptive characteristics of the study cohort (sample size = 74).

	Values (*N* = 74)	Skewness
**Demography**		
Age, years	39 (33, 43)	0.639
BMI, kg·m^−2^	26.4 (24.8, 28.6)	0.491
Hypertension	19 (26%)	1.09
**Questionnaires**		
ESS	8.0 (4.0, 11.8)	0.306
MOCA	27.0 (25.0, 29.0)	−0.432
**Polysomnography**		
Total sleep time, min	427 (386, 494)	0.043
Sleep efficiency, %	88 (83, 93)	−0.798
Latency to REM, min	88 (69, 114)	2.46
NREM 1 sleep, %	13 (7, 22)	1.22
NREM 2 sleep, %	54 (43, 59)	−0.176
NREM 1&2 sleep, %	67 (61, 75)	0.325
NREM 3 sleep, %	11 (6, 18)	0.046
REM Sleep, %	20.6 (17.7, 24.2)	−0.404
ODI, times·h^−1^	24 (11, 47)	0.617
AHI, times·h^−1^	32 (16, 51)	0.577
MinSaO₂, %	79 (69, 86)	−0.893
T90,%	4 (1, 18)	1.95
Arousal Index (Resp.), times·h^−1^	11 (4, 25)	1.38
Arousal Index (Spont.), times·h^−1^	5.9 (3.9, 9.1)	1.96

Values are display as median (Inter-quartile range). BMI, body mass index; ESS, Epworth Sleepiness Scale; MOCA, Montreal Cognitive Assessment; ODI, oxygen desaturation index; AHI, apnea-hypopnea index; MinSaO₂, Minimum pulse oxygen saturation; T90, Proportion Time with SaO₂ < 90%; Resp., respiratory; Spont., spontaneous; Prop., proportion.

### 3.2. SEM modeling

With regards to the hypoxia-related variables, the factor loading for T90% was set to 1 so that the implicit direction of the latent variable hypoxia aligns with T90% to aid interpretation, i.e., higher implicit values for hypoxia indicate more severe hypoxia. As expected, the factor loading for ODI is positive, as higher values of ODI indicate more severe hypoxia, whereas the factor loading for MinSaO₂ was negative. Similarly, the factor loading of NREM1&2 proportion was set to 1, whereas its negative correlate, NREM 3 proportion, was estimated to have a negative factor loading of −1.26.

**Figure 3:**
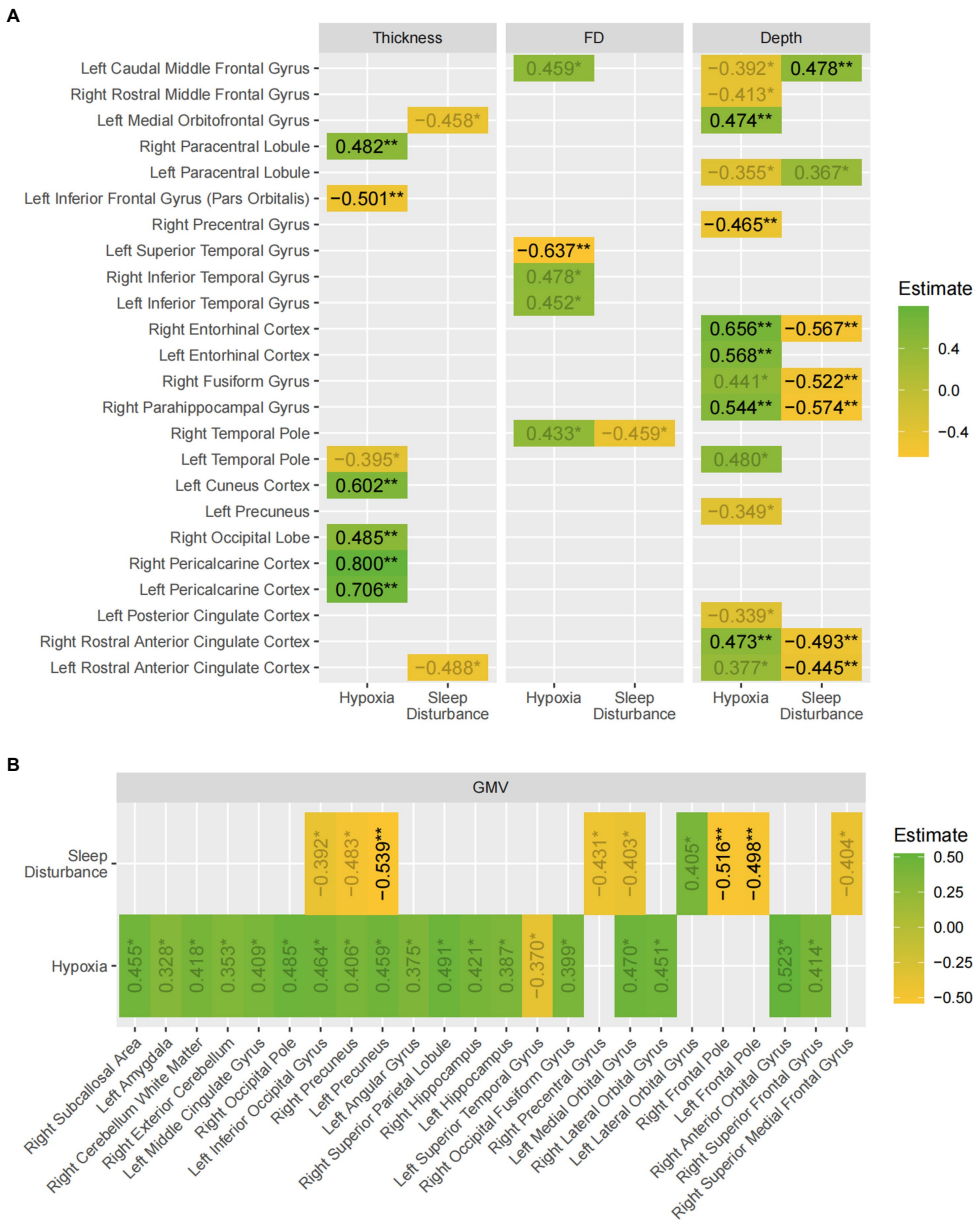
A summary of the findings of the SEM models. The values shown are the beta estimates for the latent variables, hypoxia and sleep disturbance (values with 0.01 < *p* < 0.05 are displayed in gray). Green indicates a positive estimate, whereas yellow indicates a negative estimate. **(A)** Shows the results for the three surface-based measures; **(B)** shows the results for gray matter volume. The asterisks indicate false-discovery rates: * *p* < 0.05 ** *p* < 0.01.

A total of 357 other SEM models were fitted for each structural measure in each region defined by the neuroanatomical atlases, with multiple testing corrected for using False Discovery Rate (FDR). The principal results of the SEM models are shown in Figure 3 and Supplementary Table 1, where the estimates for two latent variables regressing on the outcome measure in the main regression equation are shown alongside the FDRs. As an example, the estimates correspond to the two arrows pointing from Hypoxia and sleep disturbance to the gray matter volume of the right precuneus in Figure 2. The estimates for age were significant with FDR < 0.05 in 59 out of 358(16.5%) of the SEM models and accounting for 57 out of 209 (27.3%) ROIs in the two atlases. The estimates for age were negative for 56 out of 57 SEM models. The estimates for the other regression terms, as well as covariance relations, were similar in magnitude and same in direction as the values shown in Figure 2.

Overall, the results demonstrated widespread gray matter volume and sulcal depth differences in multiple brain regions of the frontal, parietal, and temporal lobe with hypoxia exerting statistically significant effects in more regions than sleep disturbance. Moreover, the effect of hypoxia on most regions was positive where patients experiencing worse hypoxia paradoxically exhibited higher cortical thickness, complexity, and gray matter volume. However, in most cases, the positive effects hypoxia exerted on the regions were balanced, if not inverted by the effects of sleep disturbance, as shown by the larger regression estimates for sleep disturbance in most regions with both latent variables showing significant effects.

## 4. Discussion

OSA has been known to be associated with changes in the central nervous system, leading to various cognitive impairments. For the first time, structural equation modeling (SEM) was used to analyze the effects of OSA-related hypoxia and sleep structure disruption on gray matter volume, cortical thickness, sulcal depth, and fractal dimension. The main findings of the study can be summarized as: (1) OSA exerted widespread effects on gray matter structures, and the effects were most prominent for sulcal depth and gray matter volume; (2) Overall, hypoxia tends to be associated with gray matter hypertrophy, while sleep disturbance bring about more gray matter atrophy.

In the present study, age, BMI and education were included as covariates. It is widely accepted that the brain shrinks in volume even in healthy aging, most prominently in the frontal and temporal cortex, putamen, and thalamus (Fjell and Walhovd, 2010). BMI has also been reported to modulate brain structural changes both in healthy people (Ho et al., 2011; Bolzenius et al., 2015) and OSA patients (Huang et al., 2019), and given the prevalence of obesity among OSA patients, BMI was entered as a covariate. Although the evidence on education is less well-established, some studies did report higher temporal lobe gray matter volume and reduced age-related changes associated with higher levels of education (Coffey et al., 1999; Ho et al., 2011).

One notable aspect of the results overall is that the impact of hypoxia and sleep disturbance affects the gray matter structures in no consistent manner, with a mixture of hypertrophy and atrophy in different cerebral regions. However, this is reflective of the literature on this subject, where conflicting changes were observed in individual studies showing both gray matter atrophy and hypertrophy (Baril et al., 2021), as well as between studies (Shi et al., 2017). Remarkably, some previous studies have even reported a complete absence of OSA-driven structural changes. For example, a cohort study of 312 participants showed that moderate-to-severe OSA at baseline was not associated with regional brain volume changes 15 years later (Lutsey et al., 2016), although the authors did note a possible self-selection bias where healthy participants were more likely to be present at follow-up. A meta-analysis by Shi has also highlighted the diverse range of regions where OSA-related changes were reported, including all cortical lobes and subcortical structures, though less so in the occipital cortex (Shi et al., 2017). This is in general alignment with the findings of the current study, though the last observation was not reproduced as both hypoxia and sleep disturbance were found to be significantly associated with multiple occipital structures. Checking against the several methodological pitfalls suggested by the authors of the meta-analysis to explain the apparent discordant findings, this study has kept to a high standard of robustness: (1) they suggested that some studies failed to account for important covariates such as obesity, whereas this study has included three covariates: age, BMI, and education; (2) some studies have not corrected for multiple comparisons, whereas this study used false discovery rates when reporting the SEM results.

To potentially account for this apparent lack of clear patterns of OSA-induced cortical structural alterations, a proposal was put forth by Rosenzweig et al. (2015). The authors proposed that OSA and its associated hypoxia exert its impact on the brain through a combination of adaptive and maladaptive processes, thus resulting in a mixture of gray matter hypertrophy and atrophy, and a mixture of increases and decreases in cortical thickness and complexity in different regions. The relative contribution of these processes is dependent on the dynamic interplay of various factors. Building on this, Baril et al. (2021) further hypothesized that the OSA-related brain structural changes occur in two phases. In the first phase of early and mild OSA, there are inflammatory processes lead to intracellular edema, as well as adaptive compensatory processes that leads to cortical hypertrophy, both contributing to the thickening of the gray matter regions. In the second phase, however, cortical atrophy dominates in the later and more severe patients with OSA. This hypothesis was supported primarily by an earlier study by the same group in a study of older patients with OSA (Baril et al., 2017). In that study, instead of a mixture of gray matter hypertrophy and atrophy found in other studies of younger patient cohorts, the results showed gray matter hypertrophy exclusively, in a diverse range of regions. This led to the authors’ suggestion that hypertrophy is more likely seen in studies with older participants, who tended to have just recently developed OSA and were milder in disease severity, where the earlier reactive / adaptive hypertrophic processes dominate. The current study, however, observed a widespread hypoxia-related increase in gray matter volume even in a cohort of young and middle-aged patients. As such, these results do not seem to align with the hypothesis by Cross et al. (2018) and Baril et al. (2021). Nevertheless, the dynamic interplay between adaptive and maladaptive processes, coupled with the within-study heterogeneity and cross-study differences in the patient demographic and OSA phenotypes, may offer a plausible explanation for such divergent findings between studies.

Methodologically, the SEM approach adopted by the study reduced the random measurement error by extracting the covariance of indicator variables into a combined latent variable. Arguably, this offers a more principled way to combine the various PSG-derived parameters, in contrast to the data-driven approach provided by Principal Component Analysis (PCA). The latter has been adopted by a few studies such as one by Cross et al. (2018), where the first two principal components had to be empirically labeled as “oxygen desaturation” (mainly driven by AHI, ODI, T90%, and MinSaO₂) and “sleep disturbance” (mainly driven by sleep efficiency, awakening index, and arousal index). Their results revealed a significant negative association between “oxygen desaturation” and the cortical thickness of bilateral temporal lobes, but a positive association between “sleep disturbance” and thickness in the right postcentral gyrus, pericalcarine, and pars opercularis. A similar PCA-based study by Baril et al. (2017) found a very consistent positive association between each of the three principal components (labeled as “hypoxia,” “respiratory disturbances,” and “sleep fragmentation”) and cortical thickness of various brain regions in the frontal, parietal, and cingulate cortex. Nevertheless, it should also be noted that no results were directly contradicting, i.e., opposite results in the same brain region, between this study and the two existing studies by Baril et al. (2017) and Cross et al. (2018).

Furthermore, this study was unique in that it examined gray matter cortical complexity in terms of the sulcal depth (SD) and fractal dimension (FD), which has previously rarely been studied. Unlike gray matter volume and cortical thickness, SD and FD can provide nuanced information about the shape of cortical surface. Our study found similarly mixed effects of OSA on cortical complexity, especially as measured by sulcus depth, where hypoxia was associated with increased SD in bilateral anterior cingulate cortices, bilateral rostral anterior cingulate cortices, and right parahippocampal, fusiform and entorhinal cortices and decreased SD in parts of the prefrontal and pre- and paracentral gyri. Sleep disturbance tends to be associated with the opposite effect to that of hypoxia, although in fewer regions. Along with previous studies of sulcal depth and morphological changes in aging and dementia (Kochunov et al., 2005; Im et al., 2008; Yun et al., 2013), schizophrenia (Turetsky et al., 2009) and Williams syndrome (Kippenhan et al., 2005), the results in this study suggests the utility of tapping into nuanced measures of cortical complexity to capture changes potentially overlooked by cortical thickness and gray matter volume in the OSA brain.

There are several limitations to the current study. Firstly, as the participants were drawn from a larger cohort of patients received at a hospital, this study was not able to include healthy control subjects for comparison. However, using various PSG parameters as continuous variables, the study still yielded significant findings on how disease severity moderates OSA’s effects on brain structure. As is common for studies of OSA, the duration of disease for patients was unknown, which would be an important covariate influencing the extent of OSA-associated changes, especially in teasing apart patients in the early hypertrophic phase and those in the late atrophic phase.

To conclude, the present study is the first to apply structural equation modeling to the analysis of OSA-associated brain structural changes. The results showed a significant association between hypoxia and increases in gray matter volume, cortical thickness, and cortical complexity in a diverse range of brain regions, as well as an association between altered sleep structure and decreases in the same structural measures, albeit in fewer regions. These results have highlighted the utility of the more fine-grained and advanced surface-based morphometry analysis to reveal the subtle structural differences possibly overlooked by the voxel-based measures commonly adopted by the literature. This study also served as a demonstration of a novel approach to combine the wide array of polysomnographic parameters in a unified SEM analysis of OSA-related brain structural changes.

## Data availability statement

The raw data supporting the conclusions of this article will be made available by the authors, without undue reservation.

## Ethics statement

The studies involving human participants were reviewed and approved by Research Ethics Committee of the Second Affiliated Hospital of Soochow University, Suzhou, China (JD-LK-2018-004-02). The patients/participants provided their written informed consent to participate in this study.

## Author contributions

JW and YL contributed to the study’s conception and design and data acquisition, analysis, and interpretation and drafted the paper. LJ, TS, CC, and FH contributed to data acquisition, analysis, and interpretation. EW contributed to data acquisition and analysis and revised the work critically. DC contributed to data analysis and interpretation and revised the work critically. RC contributed to the study’s conception and design and revised the work critically. All authors contributed to the article and approved the submitted version.

## Funding

This work was supported by the National Nature Science Foundation of China (grant numbers 81770085 and 82070095) and the Science, Education, and Health of Suzhou Youth Science and Technology Project (grant number KJXW2021016).

## Conflict of interest

The authors declare that the research was conducted in the absence of any commercial or financial relationships that could be construed as a potential conflict of interest.

## Publisher’s note

All claims expressed in this article are solely those of the authors and do not necessarily represent those of their affiliated organizations, or those of the publisher, the editors and the reviewers. Any product that may be evaluated in this article, or claim that may be made by its manufacturer, is not guaranteed or endorsed by the publisher.

## Footnotes

^1^ www.fil.ion.ucl.ac.uk/spm

^2^ http://link.springer.com/10.1007/978-3-540-74686-7
