# Supplementary material for: The complex interplay of hypoxia and sleep disturbance in gray matter structure alterations in obstructive sleep apnea patients
Source: Front Aging Neurosci. 2023 Mar 31;15:1090547. doi: 10.3389/fnagi.2023.1090547 (PMC10102425; doi:10.3389/fnagi.2023.1090547)
Supplement: Supplementary file 1 [file Table_1.DOCX]

Supplementary Material

# Supplementary Table

e-Table 1 Results of the SEM models, showing only the regions where at least one of the two latent variables had a significant effect on the outcome measure

|  | Hypoxia |  | Light Sleep |  |
| --- | --- | --- | --- | --- |
| Region | Estimate | FDR | Estimate | FDR |
| **Cortical Thickness** |  |  |  |  |
| Left Medial Orbitofrontal Gyrus | 0.277 (-0.002, 0.556) | 0.206 | -0.458 (-0.744, -0.172) | 0.037* |
| Right Paracentral Lobule | 0.482 (0.212, 0.753) | 0.004** | -0.357 (-0.615, -0.100) | 0.072 |
| Left Inferior Frontal Gyrus (Pars Orbitalis) | -0.501 (-0.772, -0.230) | 0.003** | 0.225 (-0.034, 0.484) | 0.269 |
| Left Temporal Pole | -0.395 (-0.675, -0.115) | 0.036* | 0.115 (-0.152, 0.381) | 0.619 |
| Left Cuneus Cortex | 0.602 (0.335, 0.870) | <0.001** | -0.262 (-0.517, -0.006) | 0.233 |
| Right Occipital Lobe | 0.485 (0.198, 0.773) | 0.007** | -0.298 (-0.579, -0.016) | 0.233 |
| Right Pericalcarine Cortex | 0.800 (0.538, 1.062) | <0.001** | -0.315 (-0.563, -0.067) | 0.112 |
| Left Pericalcarine Cortex | 0.706 (0.439, 0.973) | <0.001** | -0.361 (-0.616, -0.107) | 0.072 |
| Left Rostral Anterior Cingulate Cortex | 0.127 (-0.146, 0.400) | 0.432 | -0.488 (-0.757, -0.218) | 0.017* |
| **Fractal Dimension** |  |  |  |  |
| Left Caudal Middle Frontal Gyrus | 0.459 (0.163, 0.755) | 0.022* | -0.194 (-0.461, 0.072) | 0.371 |
| Left Superior Temporal Gyrus | -0.637 (-0.936, -0.338) | 0.001** | 0.315 (0.045, 0.584) | 0.290 |
| Right Inferior Temporal Gyrus | 0.478 (0.195, 0.761) | 0.016* | -0.250 (-0.525, 0.025) | 0.290 |
| Left Inferior Temporal Gyrus | 0.452 (0.175, 0.728) | 0.016* | -0.244 (-0.508, 0.020) | 0.290 |
| Right Temporal Pole | 0.433 (0.169, 0.698) | 0.016* | -0.459 (-0.730, -0.189) | 0.039* |
| **Sulcal Depth** |  |  |  |  |
| Left Caudal Middle Frontal Gyrus | -0.392 (-0.659, -0.126) | 0.018* | 0.478 (0.203, 0.752) | 0.006** |
| Right Rostral Middle Frontal Gyrus | -0.413 (-0.695, -0.130) | 0.018* | 0.295 (0.020, 0.571) | 0.175 |
| Left Medial Orbitofrontal Gyrus | 0.474 (0.193, 0.755) | 0.007** | 0.029 (-0.230, 0.289) | 0.846 |
| Left Paracentral Lobule | -0.355 (-0.623, -0.087) | 0.034* | 0.367 (0.100, 0.635) | 0.044* |
| Right Precentral Gyrus | -0.465 (-0.741, -0.189) | 0.007** | 0.185 (-0.082, 0.453) | 0.425 |
| Right Entorhinal Cortex | 0.656 (0.371, 0.941) | <0.001** | -0.567 (-0.835, -0.298) | 0.001** |
| Left Entorhinal Cortex | 0.568 (0.264, 0.872) | 0.004** | -0.119 (-0.389, 0.151) | 0.549 |
| Right Fusiform Gyrus | 0.441 (0.148, 0.734) | 0.017* | -0.522 (-0.797, -0.247) | 0.003** |
| Right Parahippocampal Gyrus | 0.544 (0.278, 0.809) | 0.001** | -0.574 (-0.843, -0.305) | 0.001** |
| Left Temporal Pole | 0.480 (0.176, 0.785) | 0.013* | -0.142 (-0.418, 0.134) | 0.492 |
| Left Precuneus | -0.349 (-0.625, -0.074) | 0.044* | 0.174 (-0.096, 0.443) | 0.430 |
| Left Posterior Cingulate Cortex | -0.339 (-0.612, -0.066) | 0.047* | 0.149 (-0.107, 0.405) | 0.430 |
| Right Rostral Anterior Cingulate Cortex | 0.473 (0.196, 0.750) | 0.007** | -0.493 (-0.782, -0.203) | 0.006** |
| Left Rostral Anterior Cingulate Cortex | 0.377 (0.094, 0.659) | 0.034* | -0.445 (-0.700, -0.191) | 0.006** |
| **Gray Matter Volume** |  |  |  |  |
| Right Superior Medial Frontal Gyrus | 0.223 (-0.050, 0.497) | 0.182 | -0.404 (-0.670, -0.137) | 0.031* |
| Right Superior Frontal Gyrus | 0.414 (0.118, 0.710) | 0.031* | -0.308 (-0.579, -0.037) | 0.126 |
| Right Anterior Orbital Gyrus | 0.523 (0.230, 0.816) | 0.015* | -0.262 (-0.536, 0.013) | 0.182 |
| Left Frontal Pole | 0.297 (0.038, 0.557) | 0.076 | -0.498 (-0.764, -0.231) | 0.007** |
| Right Frontal Pole | 0.228 (-0.033, 0.488) | 0.150 | -0.516 (-0.789, -0.243) | 0.007** |
| Left Lateral Orbital Gyrus | -0.257 (-0.518, 0.005) | 0.114 | 0.405 (0.143, 0.667) | 0.031* |
| Right Lateral Orbital Gyrus | 0.451 (0.172, 0.730) | 0.017* | -0.246 (-0.510, 0.019) | 0.182 |
| Left Medial Orbital Gyrus | 0.470 (0.198, 0.742) | 0.015* | -0.403 (-0.669, -0.136) | 0.031* |
| Right Precentral Gyrus | 0.337 (0.068, 0.605) | 0.054 | -0.431 (-0.698, -0.163) | 0.026* |
| Right Occipital Fusiform Gyrus | 0.399 (0.114, 0.684) | 0.031* | -0.228 (-0.500, 0.044) | 0.203 |
| Left Superior Temporal Gyrus | -0.370 (-0.654, -0.086) | 0.045* | 0.306 (0.036, 0.575) | 0.126 |
| Left Hippocampus | 0.387 (0.125, 0.650) | 0.026* | -0.123 (-0.380, 0.133) | 0.468 |
| Right Hippocampus | 0.421 (0.149, 0.693) | 0.018* | -0.066 (-0.328, 0.195) | 0.686 |
| Right Superior Parietal Lobule | 0.491 (0.219, 0.764) | 0.015* | -0.336 (-0.603, -0.070) | 0.073 |
| Left Angular Gyrus | 0.375 (0.091, 0.658) | 0.043* | -0.295 (-0.569, -0.021) | 0.135 |
| Left Precuneus | 0.459 (0.178, 0.740) | 0.017* | -0.539 (-0.816, -0.263) | 0.007** |
| Right Precuneus | 0.406 (0.119, 0.693) | 0.031* | -0.483 (-0.772, -0.193) | 0.022* |
| Left Inferior Occipital Gyrus | 0.464 (0.170, 0.758) | 0.017* | -0.392 (-0.664, -0.120) | 0.042* |
| Right Occipital Pole | 0.485 (0.196, 0.774) | 0.016* | -0.243 (-0.519, 0.033) | 0.192 |
| Left Middle Cingulate Gyrus | 0.409 (0.148, 0.670) | 0.017* | -0.249 (-0.511, 0.013) | 0.182 |
| Right Exterior Cerebellum | 0.353 (0.102, 0.604) | 0.031* | -0.056 (-0.305, 0.192) | 0.716 |
| Right Cerebellum White Matter | 0.418 (0.156, 0.680) | 0.017* | -0.206 (-0.461, 0.050) | 0.215 |
| Left Amygdala | 0.328 (0.083, 0.573) | 0.041* | -0.108 (-0.348, 0.133) | 0.496 |
| Right Subcallosal Area | 0.455 (0.191, 0.719) | 0.015* | -0.247 (-0.508, 0.014) | 0.182 |
|  | | | | |

**Note:** The estimates were displayed as value (lower and upper 95% confidence interval). The asterisks indicate false-discovery rates (FDR): * *P*<0.05 ** *P*<0.01
