# Supplementary material for: The complex interplay of hypoxia and sleep disturbance in gray matter structure alterations in obstructive sleep apnea patients
Source: Front Aging Neurosci. 2023 Mar 31;15:1090547. doi: 10.3389/fnagi.2023.1090547 (PMC10102425; doi:10.3389/fnagi.2023.1090547)
Supplement: Supplementary file 2 [file Image_1.PDF]

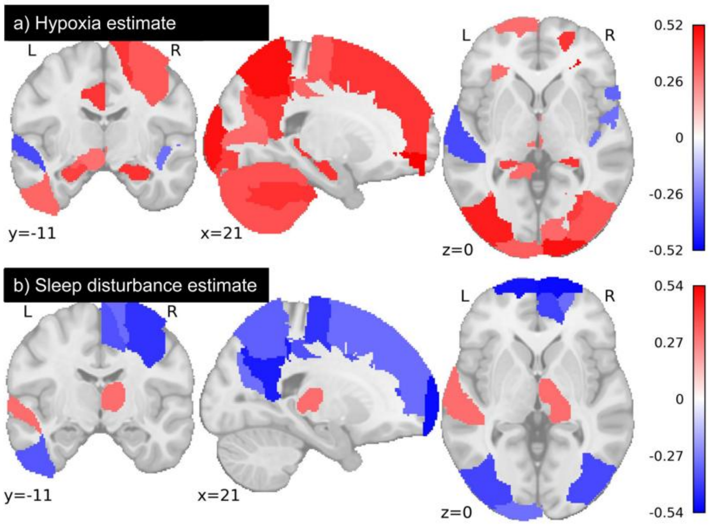

Supplement Figure 1. Changes in Gray Matter Volume. The color shown are the beta estimates in the SEM models for the latent variables, hypoxia and sleep disturbance. Red indicates a positive estimate, whereas blue indicates a negative estimate. a) shows the results for hypoxia; b) shows the results for sleep disturbance.

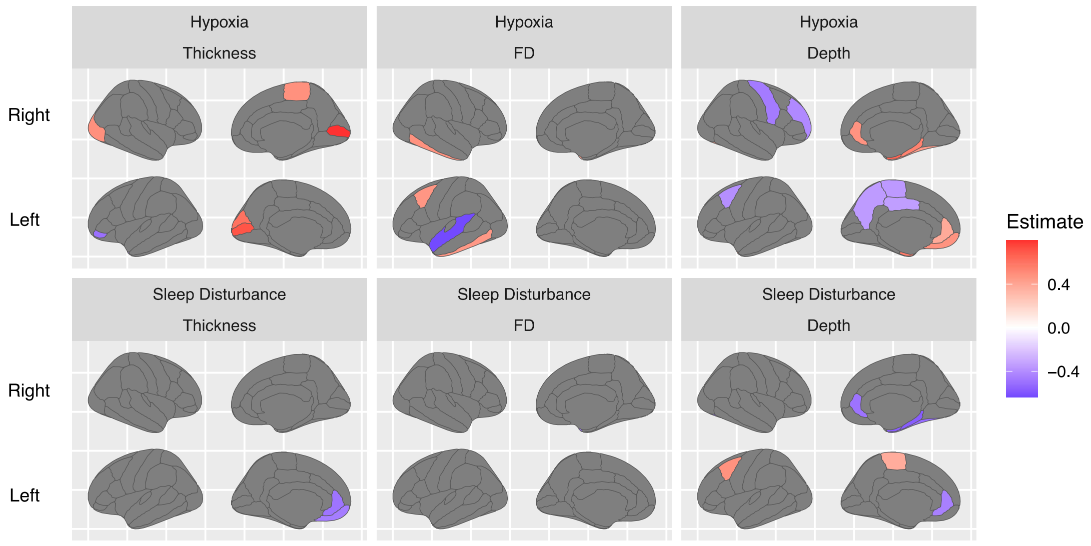

Supplement Figure 2. Changes in Thickness, Fractal Dimensions, and Depth. The color shown are the beta estimates in the SEM models for the latent variables, hypoxia and sleep disturbance. Red indicates a positive estimate, whereas blue indicates a negative estimate.

Supplement Figure 3

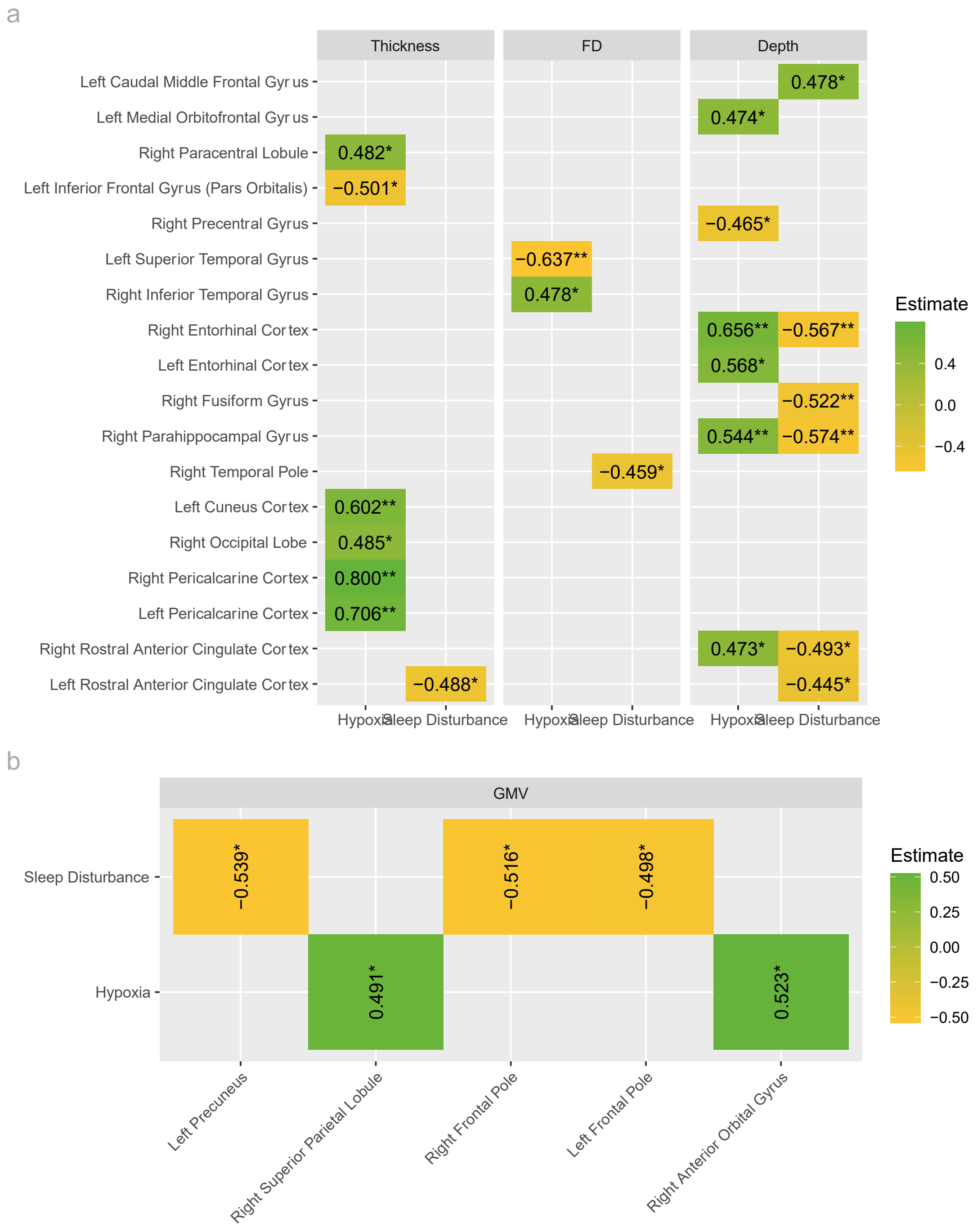

Supplement Figure 3. A summary of the findings of the SEM models adjusted using the stricter Bonferroni correction. The values shown are the beta estimates for the latent variables, hypoxia and sleep sleep disturbance (values with $0.01 < p < 0.05$ are displayed in gray). Green indicates a positive estimate, whereas yellow indicates a negative estimate. a) shows the results for the three surface-based measures; b) shows the results for gray matter volume. The asterisks indicate Bonferroni-adjusted p values: * P<0.05 ** P<0.01
